# Ambient Particulate Matter Air Pollution Exposure and Mortality in the NIH-AARP Diet and Health Cohort

**DOI:** 10.1289/ehp.1509676

**Published:** 2015-09-15

**Authors:** George D. Thurston, Jiyoung Ahn, Kevin R. Cromar, Yongzhao Shao, Harmony R. Reynolds, Michael Jerrett, Chris C. Lim, Ryan Shanley, Yikyung Park, Richard B. Hayes

**Affiliations:** 1 Department of Environmental Medicine, New York University School of Medicine, Tuxedo, New York, USA; 2 Department of Population Health, and; 3 Cardiovascular Clinical Research Center, Department of Medicine, New York University School of Medicine, New York, New York, USA; 4 School of Public Health, University of California, Berkeley, Berkeley, California, USA; 5 Division of Public Health Sciences, Department of Surgery, Washington University School of Medicine, St. Louis, Missouri, USA; 6 National Cancer Institute, National Institutes of Health, Department of Health and Human Services, Bethesda, Maryland, USA

## Abstract

**Background:**

Outdoor fine particulate matter (≤ 2.5 μm; PM2.5) has been identified as a global health threat, but the number of large U.S. prospective cohort studies with individual participant data remains limited, especially at lower recent exposures.

**Objectives:**

We aimed to test the relationship between long-term exposure PM2.5 and death risk from all nonaccidental causes, cardiovascular (CVD), and respiratory diseases in 517,041 men and women enrolled in the National Institutes of Health-AARP cohort.

**Methods:**

Individual participant data were linked with residence PM2.5 exposure estimates across the continental United States for a 2000–2009 follow-up period when matching census tract–level PM2.5 exposure data were available. Participants enrolled ranged from 50 to 71 years of age, residing in six U.S. states and two cities. Cox proportional hazard models yielded hazard ratio (HR) estimates per 10 μg/m3 of PM2.5 exposure.

**Results:**

PM2.5 exposure was significantly associated with total mortality (HR = 1.03; 95% CI: 1.00, 1.05) and CVD mortality (HR = 1.10; 95% CI: 1.05, 1.15), but the association with respiratory mortality was not statistically significant (HR = 1.05; 95% CI: 0.98, 1.13). A significant association was found with respiratory mortality only among never smokers (HR = 1.27; 95% CI: 1.03, 1.56). Associations with 10-μg/m3 PM2.5 exposures in yearly participant residential annual mean, or in metropolitan area-wide mean, were consistent with baseline exposure model results. Associations with PM2.5 were similar when adjusted for ozone exposures. Analyses of California residents alone also yielded statistically significant PM2.5 mortality HRs for total and CVD mortality.

**Conclusions:**

Long-term exposure to PM2.5 air pollution was associated with an increased risk of total and CVD mortality, providing an independent test of the PM2.5–mortality relationship in a new large U.S. prospective cohort experiencing lower post-2000 PM2.5 exposure levels.

**Citation:**

Thurston GD, Ahn J, Cromar KR, Shao Y, Reynolds HR, Jerrett M, Lim CC, Shanley R, Park Y, Hayes RB. 2016. Ambient particulate matter air pollution exposure and mortality in the NIH-AARP Diet and Health cohort. Environ Health Perspect 124:484–490; http://dx.doi.org/10.1289/ehp.1509676

## Introduction

Over the past several decades, numerous published epidemiologic studies have documented a consistent association between long-term exposure to fine particulate matter mass (≤ 2.5 μm; PM_2.5_) air pollution and an increase in the risk of mortality around the globe (e.g., Beelen et al. 2014; Brook et al. 2010; Crouse et al. 2012; Dockery et al.1993; Eftim et al. 2008; Ostro et al. 2010; Ozkaynak and Thurston 1987; Pope et al. 1995, 2002, 2004). Pope and collaborators notably found elevated relative risks of cardiovascular (CVD) mortality in association with long-term PM_2.5_ exposure [hazard ratio (HR) per 10 μg/m^3^ = 1.12; 95% confidence interval (CI): 1.08, 1.15] in the largest and most definitive U.S. nationwide cohort considered to date (Pope et al. 2002, 2004), providing a cardiovascular mortality HR of 1.12 per 10 μg/m^3^ (95% CI: 1.08,1.15). However, existing U.S. cohort studies of PM_2.5_ health effects are still being questioned (e.g., Reis 2013). In addition, particulate matter air pollution levels have been declining in recent years in the United States, so there is a need to confirm whether studies conducted in the past at higher levels are replicable today. Thus, it is important to test these associations in another large U.S. cohort with detailed individual-level risk factor information on participants, especially one for which pollution exposures can be estimated at the individual participant residence level, and in more recent lower PM_2.5_ exposure years, as we report here. This research addresses these needs using the newly available U.S. National Institutes of Health–AARP Diet & Health cohort (NIH-AARP Study) (Schatzkin et al. 2001).

## Methods

*Study population.* The NIH-AARP Study was initiated when members of the AARP, 50–71 years of age from six U.S. states (California, Florida, Louisiana, New Jersey, North Carolina, and Pennsylvania) and two metropolitan areas (Atlanta, Georgia, and Detroit, Michigan), responded to a mailed questionnaire in 1995 and 1996. Details of the NIH-AARP Study have been described previously (Schatzkin et al. 2001). Among 566,398 participants enrolled in the NIH-AARP cohort and available for analysis in 2014, we first excluded for this analysis those individuals for whom the forms were filled out by a proxy (*n* = 15,760, or 2.8%); who moved out of their study region before January 2000 (*n* = 13,863, or 2.4%); who died before 1 January 2000 (*n* = 21,415, or 3.8%); and those for whom census-level outdoor PM_2.5_ exposure was not estimable using the methods discussed below (*n* = 737, or 0.1%). After accounting for overlapping exclusions, the analytic cohort includes 517,041 (91.3%) participants for whom matching PM_2.5_ air pollution data were available. The NIH-AARP cohort questionnaires elicited information on demographic and anthropometric characteristics, dietary intake, and numerous health-related variables (e.g., marital status, body mass index, education, race, smoking status, physical activity, and alcohol consumption) at enrollment only. Contextual environment characteristics (e.g., median income) for the census tract of each of this cohort’s participants have also been compiled for this population by the NIH-AARP Study (NIH-AARP 2006), allowing us to also incorporate contextual socioeconomic variables at the census-tract level. All participants provided informed consent before completing the study questionnaire. The study was approved by the institutional review boards of the National Cancer Institute and New York University School of Medicine.

*Cohort follow-up and mortality ascertainment.* Vital status was ascertained through a periodic linkage of the cohort to the Social Security Administration Death Master File and follow-up searches of the National Death Index Plus for participants who matched to the Social Security Administration Death Master File (unpublished data, available on request from https://www.ssa.gov/dataexchange/), cancer registry linkage, questionnaire responses, and responses to other mailings. Participants were followed for address changes using the U.S. Postal Service’s National Change of Address database, responses to other study-related mailings such as newsletters, and directly from cohort members’ notifications (Michaud et al. 2005). We used the *International Classification of Diseases, 9th Revision* (ICD-9) and the *International Statistical Classification of Diseases, 10th Revision* to define death due to CVD (ICD-10: I00–I99), nonmalignant respiratory disease (ICD-10: J00–J99), and deaths from nonexternal and nonaccidental deaths (ICD-10 A00–R99). During the follow-up period considered here (2000 through 2009), 86,864 (16.8%) participants died, of whom 84,404 (97.2% of deaths) participants died of nonexternal and nonaccidental causes.

**Figure 1:**
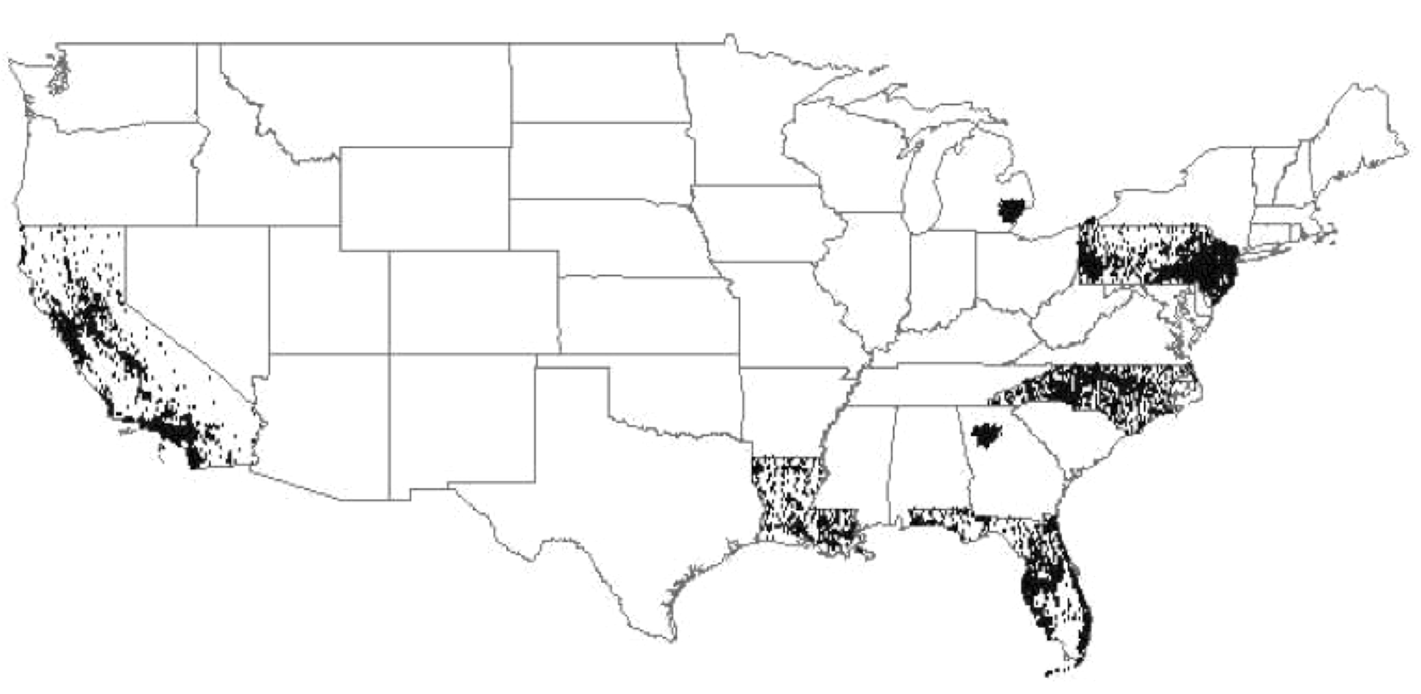
Continental U.S. map of NIH-AARP study participants’ census tracts.

*Air pollution exposure assessment.* Outdoor annual PM_2.5_-related exposures at the census-tract level for residences at NIH-AARP cohort entry were estimated using data from the U.S. Environmental Protection Agency (EPA) nationwide Air Quality System (AQS, formerly AIRS) (http://www.epa.gov/airdata/). The nationwide AQS Network includes nearly 3,000 sites, has operated since the 1970s, and has included measurement of PM_2.5_ mass since mid-1999. The year 2000 was selected as the start of follow-up in this study because that is the first full year that outdoor PM_2.5_ exposure data were available nationwide. The contiguous U.S. map in Figure 1 displays the census tracts in which the members of this cohort resided at the start of the study. Private residence locations were not included in the original NIH-AARP Cohort data set in order to protect participant privacy. As a result, we employed census tract centroid estimates of monthly average PM_2.5_ mass exposures available through the year 2008, as obtained from a published hybrid land-use regression (LUR) geostatistical model (Beckerman et al. 2013), and as matched with individuals by NIH to further protect participant anonymity. Exposure was considered only through 2008 because the time-dependent model matched deaths with exposure in each prior year, and follow-up ended in 2009 for these analyses. These estimates used ambient AQS PM_2.5_ as the dependent variable and traffic and land use information as predictors (Beckerman et al. 2013). Residuals from this model were interpolated with a Bayesian maximum entropy (BME) model, and the estimates from the LUR and BME were combined post hoc to derive monthly estimates of PM_2.5_. To allow investigation of possible confounding by O_3_ exposure, annual primary metropolitan statistical area (PMSA) mean ozone (O_3_) exposures were also estimated for the year 2000 by averaging annual O_3_ means from all ambient monitoring sites with > 75% of possible days of data in each PMSA (including 391 sites among 93 PMSAs) (U.S. EPA 2014). The PMSA mean PM_2.5_ mass concentrations in 2008, at the end of the exposure period, were lower than but highly correlated with their paired PMSA mean concentration in 2000 (*R* ^2^ = 0.77), suggesting that the spatial rank ordering of PM_2.5_ concentrations remained consistent over the follow-up period. However, the number of cohort participants living below the U.S. annual PM_2.5_ standard (12 μg/m^3^) increased over time, rising steadily from only 33% of cohort participants in 2000 (mean *±* SD = 13.6 ± 3.6 μg/m^3^) up to 78% of cohort participants living below the 12 μg/m^3^ annual PM_2.5_ standard in 2008 (mean *±* SD = 10.2 ± 2.3 μg/m^3^). Therefore, to incorporate these exposure level changes over the follow-up time, we also developed annual mean exposures at the census tract centroid of each participant’s residence at baseline to incorporate into a time-dependent sensitivity analysis of the PM_2.5_–mortality association, with censoring for those known to have moved.

*Statistical methods.* Person-years of follow-up were included for each participant from 1 January 2000 to the date of death, the end of follow-up (31 December 2009), or the date the participant moved out of the state or city where s/he lived at enrollment, whichever occurred first. This period was selected because that is the time period for which outdoor PM_2.5_ exposure estimates were available nationwide at the census-tract level for matching with the cohort mortality data (Beckerman et al. 2013). For the time-independent exposure model, the exposure metric was each participants’s annual mean enrollment census tract–centroid PM_2.5_ exposure in the first year of this mortality analysis, 2000, which was the first complete year of PM_2.5_ data availability across the United States. In addition, we also considered a time-dependent (annual mean) model, for which annual mean census tract–level exposure to PM_2.5_ was treated as time-varying, with a 1-year lag. For example, mortality risk during 2000 was related to each participant’s enrollment residence census tract–specific average PM_2.5_ for 1999.

We used the Cox proportional hazards models (Cox and Oakes 1984; Fleming and Harrington 1991) to estimate relative risks (RRs) of mortality and 95% confidence intervals (CIs) in relation to ambient PM_2.5_ (per 10 μg/m^3^). In multivariate models including individual-level variables, we treated age (in 3-year groupings), sex and region (six U.S. States and two municipalities of residence at study entry) as strata and adjusted for the following individual covariates and potential risk factors at enrollment: race (non-Hispanic white, non-Hispanic black, other), education (< 8 years, 8–11 years, high school, some college, college graduate), marital status (married, never married, or other, including widowed/divorced/separated and unknown), body mass index (BMI; < 18.5, 18.5 to < 25.0, 25.0 to < 30.0, 30 to < 35, and ≥ 35 kg/m^2^), alcohol consumption (none, < 1, 1–2, 2–5, and ≥ 5 drinks per day), and smoking history (never smoker, former smoker who quit at least 1 year ago of ≤ 1 pack/day, former smoker who quit at least 1 years ago of > 1 pack/day, quit less than 1 year ago or current smoker of ≤ 1 pack/day, quit less than 1 year ago or current smoker of > 1 pack/day). We also included two contextual characteristics of the participants’ residential census tracts found to modify the PM_2.5_–mortality HR estimates and have statistical significance in our analyses (data not shown): *a*) median census tract household income; and *b*) percent of census tract population with less than a high school education, based on the 2000 decennial census for the residence at study entry, as included in the cohort data set (NIH-AARP 2006). Potential effect modification was assessed by including multiplicative interaction terms between PM_2.5_ concentrations and each covariate [e.g., sex, age < 65 or ≥ 65 years, age and sex combined, education (< high school, high school, > high school), and smoking (never, former, current) at baseline] in the proportional hazards models. Likelihood ratio statistic *p*-values (two-sided) comparing model fit with and without interaction terms were used to test the statistical significance of each interaction, with *p*-values of < 0.05 defined as statistically significant. Statistical analyses were carried out in SAS (version 9.3; SAS Institute Inc.) and R (version 3.0.1), using the “survival” package (R Core Team 2013).

Additional sensitivity analyses were conducted, including models without adjusting for contextual variables; limiting the analysis to California residents; without censoring data after people moved; adjusting for O_3_, and using PM_2.5_ exposures estimated at the metropolitan area average level (rather than at the census tract level). In addition, other contextual characteristics were also considered: *a*) Gini coefficient, a metric of income inequality; *b*) percent of census tract population who are black; *c*) percent of census tract population who are unemployed; and *d*) percent of census tract population living below the poverty level, but were not included in the final model, as addition of these variables did not significantly affect results. To allow more direct comparisons with past work applying random effects methods (e.g., Krewski et al. 2009), we also evaluated HRs in relation to baseline (2000) PM_2.5_ exposure levels while incorporating random effects for state of residence using the “coxme” package in R.

To show how the shape of the PM_2.5_–mortality relationship response varies with concentration in this cohort, PM_2.5_ natural spline (ns) plots with 4 degrees of freedom (df) were prepared for both total (all cause) and cardiovascular mortality using standard Cox models for the baseline case, stratified by age and sex, and adjusted for all individual-level covariates and contextual variables, as described above.

## Results

The cohort was exposed to a wide range of PM_2.5_ concentrations (Table 1), with a concentration range similar to the nation as a whole (U.S. EPA 2009). Except for race (for which Table 1 indicates a rising exposure with increasing percentage of black participants), cohort characteristics were generally similar across PM_2.5_ exposure level, limiting the potential for confounding in our PM_2.5_ mortality relationship analyses.

**Table 1:** Selected participant characteristics according to quintile of PM_2.5_ exposure in 2000 [mean ± SD or *n* (%)].

Characteristic	PM_2.5_ concentration (μg/m^3^)				
	2.9–10.7	10.7–12.6	12.6–14.2	14.2–15.9	15.9–28.0
*n*^*a*^	103,576	103,330	103,345	103,410	103,380
Age in 2000 (years)	66.1 ± 5.3	65.8 ± (5.4)	65.6 ± (5.4)	65.6 ± (5.4)	65.6 ± (5.4)
Sex
Male	60,996 (58.9)	61,716 (59.7)	61,541 (59.5)	61,076 (59.1)	58,053 (56.2)
Female	42,580 (41.1)	41,614 (40.3)	41,804 (40.5)	42,334 (40.9)	45,327 (43.8)
BMI (kg/m^2^)
≤ 18.5	845 (0.8)	817 (0.8)	842 (0.8)	809 (0.8)	860 (0.8)
18.5–25	37,390 (36.1)	34,657 (33.5)	33,316 (32.2)	32,861 (31.8)	35,545 (34.4)
> 25 and ≤ 30	42,709 (41.2)	43,141 (41.8)	43,329 (41.9)	43,327 (41.9)	41,781 (40.4)
> 30 and ≤ 35	14,714 (14.2)	15,959 (15.4)	16,546 (16.0)	16,794 (16.2)	15,823 (15.3)
> 35	5,329 (5.1)	6,041 (5.8)	6,510 (6.3)	6,816 (6.6)	6,531 (6.3)
Unknown	2,589 (2.5)	2,715 (2.6)	2,802 (2.7)	2,803 (2.7)	2,840 (2.7)
Smoking status
Never smoking	34,685 (33.5)	35,363 (34.2)	37,100 (35.9)	37,413 (36.2)	38,377 (37.1)
Former, ≤ 1 pack/day	28,700 (27.7)	27,572 (26.7)	27,307 (26.4)	27,219 (26.3)	27,442 (26.5)
Former, > 1 pack/day	23,163 (22.4)	22,575 (21.8)	21,285 (20.6)	20,414 (19.7)	19,696 (19.1)
Currently, ≤ 1 pack/day	8,555 (8.3)	8,709 (8.4)	8,855 (8.6)	9,541 (9.2)	9,368 (9.1)
Currently, > 1 pack/day	4,657 (4.5)	5,232 (5.1)	4,895 (4.7)	4,812 (4.7)	4,543 (4.4)
Unknown	3,816 (3.7)	3,879 (3.8)	3,903 (3.8)	4,011 (3.9)	3,954 (3.8)
Race/ethnicity
White	95,786 (92.5)	95,942 (92.9)	96,283 (93.2)	94,670 (91.5)	88,741 (85.8)
Black	1,807 (1.7)	2,501 (2.4)	3,532 (3.4)	5,421 (5.2)	7,067 (6.8)
Hispanic	2,691 (2.6)	1,974 (1.9)	1,180 (1.1)	920 (0.9)	3,011 (2.9)
Asian	1,957 (1.9)	1,573 (1.5)	1,004 (1.0)	1,043 (1.0)	2,863 (2.8)
Unknown	1,335 (1.3)	1,340 (1.3)	1,346 (1.3)	1,356 (1.3)	1,698 (1.6)
Marital status
Married	71,327 (68.9)	72,457 (70.1)	72,094 (69.8)	70,980 (68.6)	65,450 (63.3)
Widowed/divorced/separated	26,664 (25.7)	25,923 (25.1)	25,816 (25.0)	26,592 (25.7)	30,330 (29.3)
Never married	4,743 (4.6)	4,135 (4.0)	4,563 (4.4)	5,019 (4.9)	6,646 (6.4)
Unknown	842 (0.8)	815 (0.8)	872 (0.8)	819 (0.8)	954 (0.9)
Education
Less than 11 years	5,081 (4.9)	6,011 (5.8)	6,829 (6.6)	7,198 (7.0)	5,672 (5.5)
High school completed	17,019 (16.4)	19,880 (19.2)	22,604 (21.9)	24,055 (23.3)	17,750 (17.2)
Post–high school	9,560 (9.2)	10,590 (10.2)	10,652 (10.3)	10,933 (10.6)	8,890 (8.6)
Some college	25,852 (25.0)	24,470 (23.7)	21,809 (21.1)	21,616 (20.9)	25,854 (25.0)
College and post graduate	43,103 (41.6)	39,343 (38.1)	38,347 (37.1)	36,498 (35.3)	42,001 (40.6)
Unknown	2,961 (2.9)	3,036 (2.9)	3,104 (3.0)	3,110 (3.0)	3,213 (3.1)
State of residence
California	49,086 (47.4)	26,087 (25.2)	12,303 (11.9)	13,238 (12.8)	59,495 (57.5)
Florida	47,001 (45.4)	42,769 (41.4)	14,647 (14.2)	5,851 (5.7)	82 (0.1)
Georgia	0 (0.0)	0 (0.0)	0 ( 0.0)	156 (0.2)	14,331 (13.9)
Louisiana	265 (0.3)	3,717 (3.6)	12,150 (11.8)	3,295 (3.2)	145 (0.1)
Michigan	78 (0.1)	1,157 (1.1)	3,051 (3.0)	15,546 (15.0)	6,307 (6.1)
North Carolina	156 (0.2)	8,022 (7.8)	11,596 (11.2)	18,402 (17.8)	4,583 (4.4)
New Jersey	4,585 (4.4)	14,568 (14.1)	29,238 (28.3)	14,657 (14.2)	2,149 (2.1)
Pennsylvania	2,405 (2.3)	7,010 (6.8)	20,360 (19.7)	32,265 (31.2)	16,288 (15.8)
Contextual variables
Median income ($)	57,399 ± 27,037	52,980 ± 23,695	53,453 ± 22,793	51,280 ± 20,502	53,746 ± 22,979
Percent high school or less	13.6 ± 9.6	15.5 ± 10.0	15.6 ± 9.7	16.2 ± 9.8	18.0 ± 13.7
^***a***^Number of participants in PM_2.5_ quintile, after accounting for missing covariate data.					

**Figure 2:**
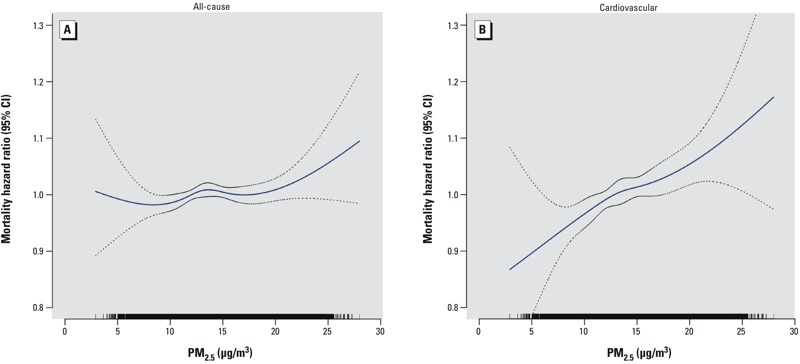
Concentration–response curves (solid lines) and 95% CIs (dashed lines) based on natural spline models with 4 df, standard Cox models stratified by age and sex, adjusted for all individual-level covariates (race, education, marital status, BMI, alcohol consumption, and smoking history) and contextual covariates [median income ($), and percent high school or less] for (*A*) all nonaccidental causes and (*B*) cardiovascular disease. The tick marks on the *x*-axis identify the distribution of observations according to PM_2.5_ concentrations.

In our time-independent baseline exposure Cox model analyses of the selected cohort (using the study entry tract of residence PM_2.5_ mean as the exposure reference for each participant), higher levels of ambient PM_2.5_ exposure were significantly associated with increased mortality due to all causes of (nonaccidental) death (HR = 1.03 per 10 μg/m^3^ PM_2.5_; 95% CI: 1.00, 1.05) and cardiovascular disease (HR = 1.10; 95% CI: 1.05, 1.15), as presented in Table 2. Stratified analyses by sex, age, and education for this cohort did not indicate significant differences in PM_2.5_ effect estimates across categories (Table 2). However, although PM_2.5_ exposure was not significantly associated overall with increased risk of respiratory mortality (HR = 1.05; 95% CI: 0.98, 1.13), an association was found for never smokers (HR = 1.27; 95% CI: 1.03, 1.56). Figure 2 graphically demonstrates, for the time-independent model, the monotonically rising nature of the concentration–response curve for both all-cause and CVD mortality (vs. a referent HR = 1.0 at the mean level of exposure).

**Table 2:** NIH-AARP cohort time independent Cox model PM_2.5_ mortality hazard ratios (and 95% CIs) per 10 μg/m^3^, by cause and cohort subgroup.

Cohort subset	All-cause mortality			Cardiovascular mortality			Respiratory mortality		
	HR (95% CI)	*n* deaths	*p*-int	HR (95% CI)	*n* deaths	*p*-int	HR (95% CI)	*n* deaths	*p*-int
All	1.03 (1.00, 1.05)	84,404		1.10 (1.05, 1.15)	26,009		1.05 (0.98, 1.13)	8,397
Age (years)
< 65	1.00 (0.95, 1.05)	20,422		1.09 (0.99, 1.19)	5,614		1.00 (0.85, 1.19)	1,592
≥ 65	1.03 (1.00, 1.06)	63,982	0.67	1.10 (1.05, 1.15)	20,395	0.97	1.06 (0.98, 1.15)	6,805	0.24
Sex
Male	1.03 (1.00, 1.06)	55,685		1.09 (1.04, 1.15)	18,200		1.02 (0.93, 1.12)	5,193
Female	1.02 (0.98, 1.06)	28,719	0.77	1.10 (1.02, 1.19)	7,809	0.33	1.10 (0.98, 1.23)	3,204	0.73
Sex and age (years)
Male: < 65	0.99 (0.94, 1.06)	13,117		1.08 (0.97, 1.21)	3,975		0.99 (0.80, 1.23)	923
Male: ≥ 65	1.04 (1.01, 1.08)	42,568		1.10 (1.03, 1.16)	14,225		1.03 (0.92, 1.14)	4,270
Female: < 65	1.01 (0.94, 1.10)	7,305		1.11 (0.94, 1.30)	1,639		1.01 (0.78, 1.31)	669
Female: ≥ 65	1.02 (0.97, 1.06)	21,414	0.88	1.10 (1.01, 1.19)	6,170	0.82	1.12 (0.99, 1.28)	2,535	0.56
Education
< High school education	1.02 (0.97, 1.07)	25,886		1.05 (0.97, 1.15)	8,176		1.04 (0.91, 1.19)	2,900
High school education	1.06 (0.98, 1.15)	8,668		1.21 (1.05, 1.40)	2,708		1.00 (0.79, 1.26)	883
> High school education	1.02 (0.99, 1.05)	46,577	0.65	1.10 (1.04, 1.16)	14,057	0.86	1.07 (0.97, 1.18)	4,275	0.38
Smoking
Never smoked	1.04 (0.99, 1.08)	19,785		1.11 (1.02, 1.20)	6,384		1.27 (1.03, 1.56)	1,004
Former smoker	1.02 (0.99, 1.06)	44,590		1.07 (1.01, 1.14)	13,934		1.04 (0.94, 1.14)	4,677
Current smoker	1.01 (0.95, 1.06)	16,354	0.58	1.14 (1.02, 1.25)	4,451	0.46	1.01 (0.88, 1.16)	2,372	0.70
*p*-int, *p*-value for interaction.									

A number of sensitivity analyses for alternative models were also conducted (Table 3). In general, associations were stronger and *p*-values were smaller when we did not adjust for census tract–level contextual environmental variables, including the association with respiratory mortality (HR = 1.09; 95% CI: 1.02, 1.18). Adding random-effects terms to the time-independent model yielded very similar results to those without random-effects terms. Time-dependent yearly exposure models gave comparable results to the year 2000 time-independent baseline exposure model for total mortality (HR = 1.03; 95% CI: 0.99, 1.05), CVD mortality (HR = 1.11; 95% CI: 1.06, 1.16), and respiratory mortality (HR = 1.05; 95% CI: 0.97, 1.15). Limiting the analysis to only California (the state with the largest number of cohort participants) gave similar results to the entire cohort. To assess the extent to which our censoring of those who moved out of the study state/city might have affected the results, we also present overall results for participants without that censoring, retaining those who moved after 2000, finding that it gave similar results to our base model case with censoring (as shown in Table 2). In addition, in a model that simultaneously also included exposure to the gaseous pollutant O_3_ along with PM_2.5_, the PM_2.5_ effect estimate was found to be still significant and its CVD mortality effect estimate not statistically different from the model without the addition of O_3_, indicating the PM_2.5_–CVD mortality association to be robust to the addition of O_3_.

**Table 3:** NIH-AARP cohort PM_2.5_ mortality hazard ratios and 95% CIs per 10 μg/m^3^ PM_2.5_ for alternative model specifications.

Model	*n*	All	Cardiovascular	Respiratory
Full baseline model, time-independent 2000 census tract mean PM_2.5_ exposures	517,041	1.03 (1.00, 1.05)	1.10 (1.05, 1.15)	1.05 (0.98, 1.13)
Full model, time-dependent annual census tract mean PM_2.5_ exposures	517,041	1.03 (0.99, 1.05)	1.11 (1.06, 1.16)	1.05 (0.97, 1.15)
Full baseline model, 2000 PMSA mean PM_2.5_ exposures	474,565	1.01 (0.98, 1.04)	1.10 (1.04, 1.16)	1.06 (0.97, 1.16)
Full baseline model without contextual variations	517,041	1.06 (1.03, 1.08)	1.15 (1.10, 1.20)	1.09 (1.02, 1.18)
Full baseline model with random effects	517,041	1.03 (1.00, 1.05)	1.10 (1.05, 1.14)	1.06 (0.99, 1.14)
Full baseline model with O_3_	466,121	1.02 (0.99, 1.05)	1.07 (1.02, 1.12)	1.02 (0.94, 1.11)
Full baseline model retaining all who moved from study area after 2000	517,041	1.02 (1.00, 1.05)	1.10 (1.06, 1.15)	1.04 (0.97, 1.12)
Full baseline model for California only	160,209	1.02 (0.99, 1.04)	1.10 (1.05, 1.16)	1.01 (0.93, 1.10)

## Discussion

In this large prospective cohort study with detailed baseline individual-level risk factor information on study participants (e.g., smoking, BMI, alcohol use), we confirmed a monotonically increasing, and statistically significant, relationship between long-term exposure to PM_2.5_ air pollution and both all-cause and CVD mortality, even at the decreased PM_2.5_ levels experienced in the United States since 2000. Comparisons by sex, age, and education for this cohort did not indicate statistically significant differences in the mortality–PM_2.5_ association across categories.

With significant overall associations with all-cause and cardiovascular mortality, the results presented here are consistent with many, but not all, of the prior published results examining PM_2.5_ and mortality. We estimated a 3% increase (95% CI: 0, 5%) in all-cause mortality for a 10-μg/m^3^ annual increase in PM_2.5_ that, though statistically significant in this large cohort, is lower than many other past estimates. For example, a recent literature review reported a pooled effect estimate of 6% per 10 μg/m^3^ PM_2.5_ (95% CI: 4, 8%) for all-cause mortality (Hoek et al. 2013). Our overall estimate for CVD mortality (10% effect per 10 μg/m^3^; 95% CI: 5, 15%), agrees more closely with the pooled estimate for CVD mortality reported by Hoek et al. (2013) (11% per 10 μg/m^3^; 95% CI: 6, 16%).

**Figure 3:**
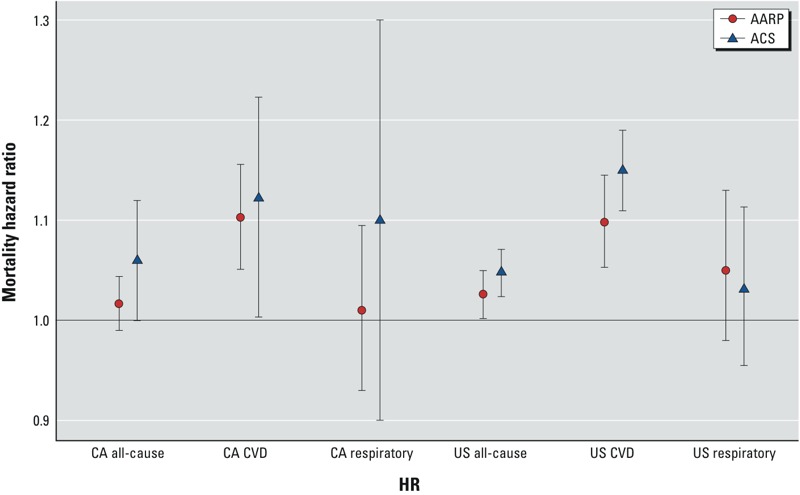
Comparison of NIH-AARP cohort vs. published ACS cohort all-cause and by-cause mortality hazard ratios per 10 μg/m^3^ PM_2.5_, with 95% CIs, for the state of California (CA) and nationwide (US) (Jerrett et al. 2013; Krewski et al. 2009).

Comparisons with the American Cancer Society (ACS) cohort, a similarly large nationwide cohort, provides an opportunity to evaluate the issue of association consistency over time in the United States. Although participants in the ACS cohort (Pope et al. 2002) were somewhat younger (mean 56 years at recruitment, vs. mean 65 years in the NIH-AARP cohort in 2000), and were exposed during that study’s follow-up to pollution at an earlier period of time (when the mix of air pollution sources was likely different), it has a similar racial (> 90% white) and educational (> 50% post–high school education) composition, is of similar size (> 500,000 participants), and also spans the United States, making it probably the most similar U.S. cohort for comparison here. The ACS cohort reported that a 10-μg/m^3^ increase in PM_2.5_ was associated with a 4% increase in all-cause mortality (95% CI: 1, 8%) (Pope et al. 2002), which is consistent with the corresponding estimate from the present analysis (3% per 10 μg/m^3^; 95% CI: 0, 5%), as shown in Figure 3. Moreover, the PM_2.5_–CVD mortality effect estimate reported for the ACS cohort (12% per 10 μg/m^3^; 95% CI: 8, 15%) (Pope et al. 2004) is very similar to the corresponding association in the NIH-AARP cohort (10% per 10 μg/m^3^; 95% CI: 5, 15%) (Figure 3). This new prospective cohort study’s follow-up begins at approximately the time that most of the published ACS cohort’s follow-up analyses ended, providing an independent test as to whether the effects continue to the lower PM_2.5_ levels in the 21st century. The ACS cohort study started in 1982 with follow-up through 1998, with an annual PM_2.5_ study period mean *±* SD = 17.7 ± 3.7 μg/m^3^ (Pope et al. 2002); in contrast, this new NIH-AARP analysis started in 2000 with much lower study follow-up mean PM_2.5_ of 12.2 ± 3.4 μg/m^3^ through 2008. Our study therefore documents for the first time that the PM_2.5_–mortality effects still occur at the much lower post-2000 levels of exposures across the United States. In California, the ACS follow-up ended with a mean 1998–2002 PM_2.5_ concentration of 14.1 μg/m^3^ (Jerrett et al. 2013), versus a much lower end of follow-up mean 2008 PM_2.5_ concentration of 10.4 μg/m^3^ in the present study. Figure 3 provides comparative plots of these two cohort’s PM_2.5_ mortality estimates across mortality outcomes, for both the United States and the State of California (Jerrett et al. 2013; Krewski et al. 2009; Pope et al. 2002, 2004), indicating consistency in their effect estimates, despite the notable decline in pollution levels after 2000.

We have also considered and compared effect estimates per 10 μg/m^3^ PM_2.5_ as a function of alternative PM_2.5_ exposure metrics. In addition to the year 2000 base PM_2.5_ exposure index, we also considered time-dependent annual mean exposure models for each mortality outcome that directly addressed the declining concentration levels of PM_2.5_ exposures during follow-up. The fixed exposure model has the advantage that it provides results using methods directly comparable to those used in many past such analyses (e.g., the ACS CP-II cohort). We found that the annual mean model yielded results consistent with the baseline (year 2000) exposure time-independent model. Lepeule et al. (2012) also found that varying the exposure metric choice had little effect on PM_2.5_ effect estimates in their analysis of the Harvard Six Cities Study cohort. Not censoring those participants who moved out of the study areas between 2000 and 2006 (*n* = 28,923) had little effect on these results. We also compared the results using both PMSA and census tract–level mean exposure metrics, finding similar and confirmatory results with either approach. This may suggest that the fact that people are mobile, and often do not stay at their home residence all day, may limit the exposure assessment accuracy gain derived from knowing home residence locale versus an area-wide average. Overall, we found that the PM_2.5_–mortality associations in this work are robust to various PM_2.5_ exposure modeling choices.

Numerous past long-term PM_2.5_–mortality analyses have found higher relative risks among those with less education. For example, Krewski et al. (2000), in their reanalysis of the Six Cities and ACS cohorts, found that the relative risk of mortality associated with fine particles was greater among individuals with high school education or less, compared to those with more than high school education in the Six Cities Study, and that the fine particle air pollution mortality risk decreased significantly (*p* < 0.05) with increasing educational attainment in the ACS cohort. They concluded that “it is possible that educational attainment is a marker for socioeconomic status, which in turn may be correlated with exposure to fine particle air pollution.” Similarly, Brunekreef et al. (2009) found in their NLCS (Netherlands Cohort Study on Diet and Cancer)–AIR cohort examination of long-term exposure to traffic air pollution that associations with mortality tended to be stronger in case–cohort participants with lower levels of education, but that differences between strata were not statistically significant. Ostro et al. (2008) also estimated stronger associations with components of PM_2.5_ among individuals with lower educational attainment, attributing this trend to the effects of lower socioeconomic status. However, no such trend was found in this NIH-AARP cohort, perhaps because the reported annual incomes of this cohort did not vary with PM_2.5_ concentration (Table 1). Indeed, although the association of education with median income in this cohort was strong (*r* = 0.49), the correlation between PM_2.5_ and median income was much lower (*p* = 0.03). Thus, it may be that the lack of a strong socioeconomic–PM_2.5_ covariation in this cohort is the reason we did not see the mortality effect modification by education status found in past studies.

This study has both strengths and limitations relative to past such studies. One strength is that we have employed estimates of PM_2.5_ exposure at the participant residence census tract level, rather than applying the overall county or metropolitan area average exposure that has been used in some major prior studies (e.g., the Medicare and ACS cohorts, respectively) (Eftim et al. 2008; Krewski et al. 2009). In addition, most previous studies have assigned only a single fixed exposure level for each study participant (e.g., at the start of the follow-up), whereas we also considered a sensitivity model applying time-varying exposure estimates to address the declining PM_2.5_ exposure levels over time. Another strength of this study is that covariate risk factors were collected at the individual level, but a limitation is that this was ascertained only at enrollment, and we could not account for temporal changes in risk factors (e.g., smoking and BMI) during follow up. Another limitation is that, other than knowing if and when participants leave the NIH-AARP cohort study areas, we presently lack information on residence location after those participants moved out of the study region. Despite these limitations, as discussed above, our derived effect estimates were found to be largely consistent with other PM_2.5_ mortality results, notably the ACS cohort study (Pope et al. 2002, 2004), the only prior prospective U.S. cohort study of such size with detailed individual-level risk factor information.

## Conclusions

Long-term exposure to PM_2.5_ air pollution was associated with a significant increase in CVD and total nonaccidental mortality in the cohort as a whole, as well as with a significant increase in respiratory mortality among never smokers, in a new, large, U.S. cohort having detailed individual level participant data and census tract–level PM_2.5_ exposure information. This independent evaluation of the PM_2.5_–mortality association, in this new large cohort, was robust to various model specification and PM_2.5_ exposure assessment sensitivity analyses, and has found effect estimates (per 10 μg/m^3^ of PM_2.5_ exposure) that are consistent with past estimates, even at the much lower PM_2.5_ air pollution levels experienced in the United States since 2000.
